# Supplementary material for: Are carbon emissions trading and green financial instruments synergistic? -Comprehensive quantitative research based on content analysis
Source: PLoS One. 2024 Mar 7;19(3):e0298601. doi: 10.1371/journal.pone.0298601 (PMC10919601; doi:10.1371/journal.pone.0298601)
Supplement: S1 Data — (ZIP) [file pone.0298601.s001.zip › Data/地级市Z_F工作报告.zip!/╣π╢1⁄2/╔ε█┌╩╨/└┤╫╘╣1⁄2╓┌║┼í╛┬φ┐╦╩2╛▌═°í┐_2003.docx]

各位代表：

　　 现在，我代表深圳市人民政府向大会作工作报告，请予审议，并请各位政协委员和其他列席人员提出意见。

　　 一、2002年的工作回顾

　　 2002年是我国改革开放和现代化建设中极不平凡的一年，党的十六大胜利召开，我国正式履行加入世贸组织的各项承诺，经济社会发展进入了一个新的阶段。在党中央、国务院、省委、省政府和中共深圳市委的正确领导下，市政府全面贯彻邓小平理论和"三个代表"重要思想，以迎接党的十六大召开和深入学习贯彻十六大精神为动力，努力把握加入世贸组织的新机遇，积极应对世界经济不景气、全球电子信息产业继续调整的国际经济环境和国内各兄弟省市加快发展、百舸争流的形势，与全市人民一道，团结拼搏、锐意进取，圆满完成了市三届人大三次会议确定的各项任务。

　　 （一）国民经济快速健康发展，发展后劲进一步增强

　　 增长速度加快，主要经济指标全面攀升。实现国内生产总值2239亿元，比上年增长15%，人均国内生产总值达4.6万元（折合5561美元），增长6.5%；工业增加值1072亿元，增长19.6%；规模以上工业总产值4221亿元（1990年不变价），增长24.2%；外贸进出口总额872亿美元，增长27.1%，其中出口总额466亿美元，增长24.2%；全社会固定资产投资747亿元，增长11%；社会消费品零售总额690亿元，增长13.2%；实际利用外资49亿美元，增长36.1%；地方财政收入304亿元，同口径比增长20.5%。

　　 经济结构优化，经济发展质量稳步提高。高新技术产业继续快速发展，实现高新技术产品产值1710亿元（现价），增长29.4%，占规模以上工业总产值（现价）的比重达47.9%。计算机、网络与通信等电子信息产业主导地位进一步强化，信息化建设和信息服务业发展步伐加快。加大了用高新技术和先进适用技术武装改造传统产业的力度，传统优势产业竞争力和科技含量进一步提高。以海港和空港运输为龙头的现代物流业蓬勃发展，深圳港集装箱吞吐量达762万标箱，增长50.1%，在全球集装箱枢纽港中的排名由2001年的第8位跃升为第6位。盐田港区三期、蛇口港区二期集装箱码头已开工建设，签定了中外合资建设大铲湾港区的协议，全面推进六大物流园区规划建设；航空货运实现了突破性发展，深圳机场货邮行吞吐量33.4万吨，增长35%，美国与韩国公司在深设立中外合资的国际航空货运和快运公司，建立了目前国内最大的波音747-400型全新货机机队，积极推进深圳机场与港澳机场的合作。金融业稳步发展，年末国内金融机构人民币各项存款余额4953亿元，增长21.1%，各项贷款余额3512亿元，增长22.8%；货币市场、外汇市场活跃，保险业持续稳健增长；创业投资资本总额约占全国的2/5，基金管理公司数量和管理的基金资产规模约占全国的一半。商贸业特别是连锁零售业发展加快，连锁企业在本市的销售额占社会消费品零售总额的36%，我市连锁企业去年在外地新开门店330家，增长2倍多，有4家连锁企业进入全国连锁业30强，其中华润万佳首次进入前10名行列，此外还动工及建成了华润新城、中信城市广场等集购物、娱乐、休闲于一体的大规模购物中心。旅游业形势继续向好，全年接待游客5000多万人次，实现旅游业总收入354亿元，其中旅游外汇收入17.2亿美元。会展业迅速兴起，成功举办了第四届"高交会"、第七届中国（深圳）国际电机电子零部件采购展、首届中国（深圳）消费商品采购大会及一大批专业展。农业稳步发展，都市型、外向型、高科技农业特色凸显。

　　 发展后劲增强，重大项目建设稳步推进。高新技术和制造业领域，去年全市已动工建设和已签订合同协议、正在做前期工作的重大项目共107个，将于今、明两年陆续建成投产，可新增工业产值3000亿元左右，再加上已有工业企业生产的正常增长，我市工业总产值到2005年可望达6200亿元以上，比2000年的2500多亿元增长1.5倍。留学生创业园、软件园、大学城、留仙洞片区、国家级集成电路设计产业化基地和研发基地等项目正按计划进行开发建设。物流与商贸服务业领域，正在建设的重大项目有11个，总投资额118亿元。基础设施领域，正在建设的重大项目有17个，总投资额681亿元。这批重大项目集中于战略支柱产业和城市基础设施，是我市经济发展后劲的重要支撑。

　　 （二）投资发展环境不断优化，对投资者和各类人才的吸引力进一步提高

　　 市委、市政府将去年确定为"优化投资发展环境年"，扎扎实实在全市范围推进全面优化投资发展环境特别是软环境的工作。政务环境方面，进一步减少行政审批事项，优化审批程序和规则，提高审批时效；继续整顿机关作风，提高行政效率和服务经济社会发展、服务企业、服务市民群众的水平；强化行政监督，加大检查落实力度，查处了一批行?"不作为"、"乱作为"的人和事，开展了"万人评议机关"活动，使机关行政效率和服务水平明显提高。法治环境方面，按照国家法制统一的原则和世贸组织规则要求，清理了149件市政府规章、2800件市政府及其部门规范性文件，配合市人大常委会初步完成了136件法规的清理工作；大力推进依法治市各方面工作，继续推进政府行政"九个法定化"，进一步提高了公务员依法行政的自觉性。营商成本方面，通过两次降低销售电价、清理行政事业性收费、撤销7个路隧收费站、取消进二线关的车辆收费等措施，全年为社会减轻负担40亿元以上。人才及生活配套服务环境方面，已建成4 所国际学校、3所港人子弟学校，规划建设2所高档次医院。全年共引进国内人才2万多人，并依托留学生创业园引进海外留学人员1000多人。市场环境方面，整顿和规范市场经济秩序、保护知识产权工作力度加大；坚持依法治税，加快推行税收现代监控征管手段，清理欠税、查处偷逃税款工作成绩显著；加快推进社会信用体系建设，企业和个人两个信用信息征集系统已投入试运行；积极推进整顿规范房地产市场秩序工作；清理违规、逾期借款和违规担保贷款工作取得成效。

　　 （三）以国有企业改革为重点的各项改革全面推进，对外开放向纵深发展

　　 国有企业改革等各项改革步伐加快。积极探索以国际招标方式进行国有企业产权转让，引进国内外战略投资者，首批5家企业试点工作进展顺利。大力推进国有企业授权经营试点工作，首批5家企业授权经营基本完成。国有企业产权主体多元化和放开搞活中小企业的工作稳步推进，共完成国有中小企业转制165户，其中整体转让或员工持股84户，停业、关闭或破产81户。通过改革，使市属国有企业资产质量明显提高，利润总额有所增长。城乡集体经济得到新发展，股份合作制等混合所有制经济不断壮大。个体、私营等非公有制经济快速发展，新注册登记私营企业17004户，增长19.3%，私营企业纳税35亿元，增长69.2%，对全市经济的贡献增大。光明农场管理体制改革和农科中心改制顺利进行。行政体制改革、投融资体制改革、政府工程建设管理体制改革、文化体制改革、统计制度改革和建立社会保障体系等其他各项改革也取得了新进展。

　　 引进外资再创佳绩。共吸引17家世界500强跨国公司来我市投资或增资，总数已达86家。引进了一批高新技术产业大项目和研发机构，如日本住友的光纤光缆项目一期等建成投产；全球第二大软件供应商甲骨文公司及瑞典爱立信公司等在深设立研发中心，英特尔公司设立了应用及设计中心。把握我国加入世贸组织机遇，金融、物流、商贸、旅游、专业服务等服务贸易领域对外开放步伐加快。沃尔玛、索尼、IBM、阿尔卡特等十多家大型跨国公司来深设立了采购中心；吉之岛、百安居等一批外资商贸企业进入深圳；美、英等国的金融保险机构与深圳的地方商业银行和保险公司签订了合资经营协议；深圳银行业实现了与香港同业间的港币支票双向结算、港币资金结算实时支付和银行卡双向结算；保税区扩区已征得海关总署等国家有关部门原则同意；等等。

　　 外贸出口持续快速增长。积极开拓多元化出口市场，进一步优化出口产品结构。高新技术产品出口157亿美元，占全市出口总额的33.7%，比上年提高4.3个百分点。一般贸易出口55.7亿美元，增长36.3%。外贸出口总额连续十年居全国大城市首位。稳步实施"走出去"战略，华为、中兴等高科技企业在境外设立了29个代表处，我市累计在境外投资的生产性和贸易性项目已达243个。保税区和出口加工区在扩大外贸出口中发挥了良好作用。积极做好"大通关"工作，改善口岸通关环境，协调推进海关监管方式改革，将EDI联网监管模式推广至近200家加工贸易企业，并实行结转深加工联网通关改革，通关效率大大提高。

　　 深港合作深入推进，皇岗口岸24小时通关顺利实施，深港大型跨界基础设施项目的协调合作取得新进展。外事、侨务、台务工作在对外开放和对外交流中发挥了积极作用。进一步加强与我国广大中西部地区和我省其他地区的合作，努力拓展国内市场，对口帮扶工作取得实效。

　　 （四）城市规划管理工作加强，基础设施建设进展良好

　　 城市规划不断完善和深化。城市规划委员会运作机制更加健全，社会各界、广大市民参与规划的积极性进一步提高。完成了高新技术产业带、卫星新城及东部海滨地区等重点规划项目，全面展开以道路交通为重点的城市基础设施规划。法定图则工作稳步推进。土地供应规模得到适度控制，房地产开发用地供应量比上年下降1/3。

　　 重大基础及配套设施建设进展顺利。地铁一期超额完成年度投资计划；新市中心区已形成各项目全面快速施工的局面，市民中心、音乐厅、博物馆、图书馆已完成土建，进入装修阶段；会展中心已完成土建基础工程；大学城各校舍单体结构已封顶；供水、供电工程建设步伐加快，东江水源第一期工程建成，年供水量达2.3亿方，岭澳核电1号、2号机组、西部电厂5号机组、深圳5号变电站、鹏城变电站提前或如期建成投入运行。

　　 城市管理工作力度加大。制定并落实关于处理历史遗留违法建筑"两个规定"的实施细则和实施办法，截止去年底共受理历史遗留违法建筑申报17.4万宗，申报率达89%；拆除各类违法建筑496万平方米，初步遏制了违法建筑泛滥的势头。注重环境污染治理和生态建设，整体环境质量维持在较好水平，全面展开了布吉河、福田河、新洲河、龙岗河、坪山河、观澜河等河流的治理污染工程。狠抓了市容环境综合整治、城市保洁和绿化美化工作。深化城市管理体制改革，在全市范围实行城市管理综合执法，建立了城管行政执法公安协同机制。实施交通综合整治，完成了若干道路交通改造工程，加强了交通管理，城市交通拥挤现象初步得到缓解。去年我市获得联合国环境规划署授予的环境保护"全球500佳"称号，并在全省城市卫生检查评比中获总分第一名。

　　 （五）各项社会事业全面进步，精神文明和民主法制建设再上新台阶

　　 精神文明建设成绩显著。在全市干部群众中认真广泛开展学习宣传邓小平理论、"三个代表"重要思想和十六大精神的活动。在全市深入进行"深圳精神如何与时俱进"大讨论，将深圳精神重新提炼概括为"开拓创新、诚信守法、务实高效、团结奉献"，振奋了人心、鼓舞了斗志。对全市党员干部进行居安思危的教育，增强了全市党员干部的忧患意识和紧迫感。深入贯彻《公民道德建设实施纲要》。群众性创建工作有声有色，涌现出一批全国先进或示范单位。"双拥"工作和国防教育、国防后备力量建设得到加强。

　　 社会主义民主法制建设不断深入。市政府自觉坚持向人大报告工作制度，自觉接受政协、民主党派和群众监督，认真办理人大议案2件、建议426件，政协建议案3件、提案529件。配合市人大加强立法工作，提请市人大常委会审议法规议案6件。颁布市政府规章16件。监察和审计部门加大了监督力度，治理"三乱"和纠正治理部门、行业不正之风工作取得新成效。进一步规范了有形建筑市场、产权交易市场和经营性土地使用权出让市场，建设工程项目优先采用经评审的低价中标法，并着力完善评标专家管理。继续强化收支两条线工作，党政机关实行了统一、规范的临时岗位津贴制度。基层民主政治稳步推进，圆满完成全市村委会换届选举工作，村务公开步入规范化、法制化轨道。推进城市基层管理体制改革，将原有443个居委会调整为351个社区。

　　 各项社会事业蓬勃发展。教育事业形势喜人，南山、罗湖、福田3个区和西乡、布吉等7个镇被评为我省"教育强区"、"教育强镇"，占全省总数的一半；全市30%的公办中学被评为省一级中学，13.5%的小学被评为省一级小学，比例在全省最高；高等教育实力大大增强，在深办学的全日制高等院校达11所，全日制高等教育毛入学率由2000年的15.7%提高为28.9%，与清华、北大、哈工大、南开等高校合作兴办的研究生院已正式招生。文化事业成果丰硕，共获国际级奖72项，国家级奖283项，举办了一系列群众喜闻乐见的文化活动，"两报"、"两台"合并顺利完成。深入推进医疗卫生体制改革，医疗服务质量稳步提高，预防保健工作得到加强。全面完成了上级下达的各项人口计划指标，全市户籍人口出生率16.6‰，计划生育率98.7%。成功承办了广东省第十一届运动会，深圳代表团夺得金牌和总分两个第二的好成绩。深圳足球队在全国甲A联赛中取得了亚军的历史最好成绩。档案工作有新进步。

　　 （六）人民生活水平进一步提高，维护社会稳定工作卓有成效

　　 居民收入稳步增加，城镇居民人均可支配收入2.49万元，增长9.6%，农民人均纯收入1.06万元，增长7.5%。居民消费结构升级步伐加快，汽车、住房等大宗商品及旅游、休闲、保健、文体等服务类消费持续增长。社会保障体系日趋完善，全市户籍职工养老保险参保率达99.8%，医疗保险参保率达98%，户籍职工和劳务工工伤保险参保率达97%，失业保险参保率达95%。加强了帮扶困难群众的工作，市委、市政府确定的57项重点帮扶项目全部落实。提高了全市最低生活保障线标准，低保对象近8000人已全部纳入最低生活保障。失业员工保险金、离退休人员养老金做到100%按时足额发放。600多户特困无房户的住房问题得到解决。法律援助机构共承办各类法律援助案件近2000件。切实加强了就业和再就业工作，近万名失业员工实现再就业，城镇居民登记失业率2.45%。第二期同富裕工程稳步推进。

　　 社会治安形势持续稳定。深入开展严打整治斗争，严厉打击"法轮功"等邪教组织，推进社会治安综合治理，确保了我市政治稳定和社会安定。全市刑事案件发案数比上年下降15.5%，破获刑事案件增加58.6%。公安机构改革及派出所整合工作基本完成，一线警力得到加强。强化了流动人口、出租屋管理和对娱乐服务场所的综合整治，有力打击了"黄、赌、毒"等社会丑恶现象。安全生产形势平稳，全年未发生重特大恶性安全事故，消防安全工作在全省评比中获得第一名。民族宗教、信访、基层调解工作在化解人民内部矛盾，维护社会稳定方面发挥了重要作用。

　　 各位代表，过去一年我市各项事业所取得的进步和成绩，是在党中央、国务院和省委、省政府及市委的正确领导下，全市人民辛勤工作、奋发图强的结果。在此，我代表市政府，向奋斗在各行各业的广大干部群众，向中央和各省驻深单位，向驻深解放军、武警官兵和各民主党派、人民团体、社会各界及外地来深建设者致以崇高的敬意！向所有参与和关心深圳建设的港澳台同胞、海外侨胞、国际友人表示衷心的感谢！

　　 在过去一年中，我们虽然取得了较大成绩，但工作中仍存在一些薄弱环节，经济社会发展中还有不少困难和问题，如：经济结构有待进一步优化，民营经济有待加快发展；行政管理体制尚须进一步创新；国有企业改革和发展的任务还很艰巨；投融资体制改革相对滞后；人口规模增长过快；城市规划、建设与管理工作跟不上城市的迅速发展，社会治安、交通状况、环境质量以及高水平的教育、医疗、卫生服务等离人民群众的要求还有一定差距；等等。对这些问题，我们一定要高度重视，采取切实措施，下大力气加以解决。

　　 二、2003年的主要工作

　　 党的十六大绘就了全面建设小康社会的宏伟蓝图，对经济特区的发展提出了新的要求。省委九届二次全会和省十届人大一次会议要求深圳加快发展，更好地发挥中心城市的作用。按照中央和省的精神，市委三届六次全会确定了今年我市工作的总体要求是：以邓小平理论?"三个代表"重要思想为指导，认真落实十六大提出的各项任务，以"增创新优势，更上一层楼，率先基本实现社会主义现代化"为总目标总任务统揽全局，围绕建设现代化中心城市和国际化城市的定位要求，努力建设高科技城市、现代物流枢纽城市、区域性的金融中心城市、美丽的海滨旅游城市、高品位的文化和生态城市，在新的起点上开创深圳改革开放和现代化建设的新局面。

　　 根据市委总体工作要求，综合考虑各方面因素，按照积极稳妥、留有余地的原则，建议我市今年的主要预期经济指标定为：国内生产总值2520亿元，增长12%；城镇登记失业率控制在3%以内；居民消费价格总指数102%；进出口总额960亿美元，增长10%，其中出口总额512亿美元，增长10%；工业增加值1215亿元，增长14.5%；规模以上工业总产值（1990年价）4940亿元，增长17%；全社会固定资产投资815亿元，增长9%；社会消费品零售总额773亿元，增长12%；实际利用外资略有增长；地方财政收入同口径增长12%。

　　 为完成上述目标任务，今年着重抓好以下几个方面的工作：

　　 （一）以优化整体经济素质、增强发展后劲、提升国际竞争力为核心，加快推进经济结构的战略性调整

　　 继续突出发展高新技术产业。努力推动我市高新技术产业由加工装配为主向自主研发、设计、制造转型，提高核心竞争力。把握世界电子信息产业结构调整机遇，在快速增长的移动通讯、笔记本电脑、光显示器件、数字化视听产品等领域，积极争取引进、新上一批大项目；在有效规避投资风险与市场风险的基础上，抓好大规模集成电路、光通信产业大项目建设； 加快发展计算机与网络服务业，引进和培育一批大型软件企业，加快软件园区建设。在生物技术、新材料、新能源、光机电一体化等新兴高新技术产业领域抓紧引进或新上一批研发机构和产业化项目，重点发展基因诊断治疗、生物芯片、转基因动植物等关键技术的应用与产业化，加快发展纳米碳管和新型动力电池产业，努力扩大生物制药、医疗器械等行业的优势。加快高新技术产业带特别是留仙洞片区、大工业区、宝龙片区的规划建设，全面启动光明南片区建设，形成高新技术企业的聚集效应?"扎根"效应。加快建立地区技术创新体系，着力提高科技创新能力，集中力量搞好12项重大科技专项；争取申报1-2家国家级工程技术研发中心，搞好3-5个重点实验室和一批公共技术平台建设；完善科技成果转化和扩散机制；鼓励和扶持创业投资，健全创业服务体系；大力实施专利和技术标准战略；大力吸引留学生来深创办高科技企业，加快建设留学生创业大厦和留学生公寓。

　　 继续加快发展现代服务业。一是加快建设面向国内外两个市场的物流中心。加快推进盐田港区三期A、蛇口港区二期、赤湾港区12、13号泊位、六大物流园区等重大物流基础设施建设，完成机场候机楼改造，抓紧做好大铲湾港区、盐田港区三期B、蛇口港区三期、深圳机场第二跑道等项目的规划及前期准备工作。继续大力发展港口集装箱运输和航空货运。依托物流园区和保税区，鼓励支持专业物流企业发展，吸引跨国公司和香港物流企业来我市开展物流业务。二是高度重视金融业发展，增强我市金融业竞争力、吸引力，巩固区域性金融中心地位。出台鼓励扶持金融业发展的政策措施。成立金融综合协调服务机构。加快金融业对外开放，加强与香港金融业的合作，强化与香港金融市场紧密联结的特色。积极支持金融业进行体制与机制、产品和服务创新，营造与国际商务接轨的金融服务环境。加快金融中心区规划建设和金融信息化工程建设步伐，形成良好的聚集效应。尽力争取创业板或其他创新业务早日推出。支持深交所扩大国债交易规模。三是继续加快商贸业发展。推进市中心区商贸项目和华润新城购物广场建设，启动人民南精品商业街、南山中心商业区等重点商业网点建设项目；加快建设电子产品、汽车展示与交易、汽车零部件、工业原材料、建筑装饰材料等一批中高级批发市场，使我市成为区域性的购物中心和批发中心。鼓励发展连锁经营，促其向更广的服务领域和市场空间拓展。四是以创?"中国最佳旅游城市"为契机，进一步改善旅游环境，推动旅游业上新台阶。积极推进与香港旅游业合作，形成联结国内外旅游市场的特色。加快东部黄金海岸和深圳湾填海区的规划、设计和建设，搞好三洲田综合生态旅游项目，形成海滨旅游城市的亮丽风景线。五是大力发展会展业。政府支持与市场化运作相结合，培育若干知名会展品牌?"高交会"和消费商品采购大会等一批重要展会要办出更大影响，做到月月有大展，常年展不断。六是加快发展信息服务、中介服务、文化产业、社区服务业等新型服务业，使之成为我市经济的新增长点。

　　 积极发展高附加值的先进制造业。根据自身的基础和条件，积极争取发展以光机电一体化为方向的装备产业、汽车零部件产业、环保设备产业等，着手规划新上一批精细化工项目，扩大集装箱、登机桥、模具、精密仪器仪表等制造业的规模，拓展我市制造业的发展空间。

　　 加大用高新技术和先进适用技术武装、改造传统产业的力度。引导我市钟表、家具、玩具、服装、珠宝、印刷、皮革等行业向加工与设计相结合转变，提高产品技术水平和附加值。组建2-3个优势传统行业的公共技术平台，筹建工业设计院和材料表面分析测试中心，推动行业技术升级和产品更新换代。引导更多企业采用CAD/CAM技术，着力提高企业设计水平。鼓励更多有条件的企业加大技改投资力度，创建技术中心。此外，要加快农业产业结构调整步伐，大力发展现代农业，积极推进公明、光明生态农业和东部海洋生物高新技术产业片区建设。

　　 加快推进信息化建设。信息化建设是我们走新型工业化道路、不断提高服务业现代化水平的关键环节。要继续搞好信息基础设施建设，今年基本建成具有国际先进水平的宽带城域网和宽带接入网。推动企业信息化建设，提高企业竞争力和经营管理水平。完善电子商务综合环境，推动电子商务的应用。积极推进覆盖全社会各领域的信息应用系统建设，不断提高城市信息化水平。

　　 大力推进企业结构调整，积极培育创新能力和核心竞争力较强的企业群体。深圳要提高国际竞争力，必须有一批技术先进、规模较大的企业作为依托和支撑。因此，我们要遵循市场经济规律，引导、推动、支持做大做强一批主业集中、主导产品突出、掌握核心技术、拥有自主知识产权和知名品牌的大企业，使之成为深圳经济的主力舰队。支持发展若干具有国际竞争力的大企业集团，培育我们自己的跨国公司。要把实施名牌战略与大企业战略有机结合起来，依托大企业在我市各主要产业培育一批知名品牌。引导、支持中小企业的发展，构筑专业化分工协作体系。努力在我市若干具有战略优势的产业领域，形成以具有较大规模和实力的大企业为龙头，大批体制机制灵活、技术创新能力强的中小企业为之配套的企业群体。

　　 （二）努力扩大出口、投资和消费，拉动经济快速增长

　　 千方百计扩大外贸出口。一是进一步加大开拓国际市场力度。继续实施"市场多元化"和"科技兴贸"战略。提升加工贸易水平，努力扩大一般贸易出口，积极拓展服务贸易、技术贸易。继续做好进出口经营权核准制改革和外商从事一般贸易改革试点工作，争取设立更多的中外合资外贸企业。支持我市有条件的企业走向国际市场，参与国际合作与竞争，鼓励和支持我市通信、电子等优势企业到国外设立研发中心、生产基地、销售网络，开展跨国经营。继续争取更多跨国公司采购中心落户深圳，今年力争总数超过30家，使我市逐步成为一个国际性的采购中心。二是改善外贸出口环境。加强外贸出口中介组织建设，完善外贸服务平台和服务体系。帮助和鼓励企业用?"免、抵、退"政策，积极协调做好出口退税工作。推进保税区综合改革，做好保税区扩区工作。加快出口加工区发展。推进大通关进一步"提速"，继续推广EDI联网监管模式，完善便捷通关措施。抓紧建立反倾销快速反应机制和外贸法律援助体系。

　　 进一步加大固定资产投资力度。继续保持较大强度的政府投资，市、区两级政府共投入200亿元，重点投向城市基础设施和社会公益领域的项目，主要包括：改善城市交通方面的项目，如地铁一期、宝松快速干道、盐坝高速公路、盐排高速公路、深平快速干道等，地铁二期、轻轨要做好前期工作；高新技术产业带和重大物流基础设施项目；土地开发项目，如深圳湾填海区、前海填海区、龙华拓展区、条件成熟的三、四个卫星新城及各区的重点工业和物流园区等；能源和环保项目，如西部电厂6号机组、深圳电网18项110千伏及以上电压输变电工程、深圳河治理三期工程等； 新市中心区项目群及一批教育、医疗卫生、文化基础设施，如会展中心、市民中心、图书新城、音乐厅、博物馆等，规划设计具有国际先进水平的新大剧院。要切实抓紧和推进今年列入计划的重大项目建设，市、区有关部门和单位全力以赴、通力合作，形成推动重大项目建设的合力。设立重大项目建设"绿色通道"，在依法规范行政的同时，简化审批程序和办事环节，大大提高审批时效。建立健全重大项目储备库，加快培育一批具有战略意义的重大项目，深化、细化前期准备工作。

　　 努力扩大消费需求，积极开拓国内市场。积极探索以大物流企业、连锁企业和中高级批发市场为纽带，把深圳经济辐射的腹地拓展至全省及华南、西南、华中等地区，带动我市产品销售，提升深圳物流枢纽地位。以大流通拓展深圳经济发展空间，认真策划组织好在我市举办的各种产品展销会和采购大会，组织企业参加在全国各地举办的大型专业展销会、贸易洽谈会。引导部分外向型企业以品牌产品拓展国内市场。繁荣本地消费，加快建设大型综合商城，营造更加良好的消费环境，吸引更多游客来深旅游、消费。合理引导住宅、汽车等大宗消费，挖掘和培育信息服务、文化、教育、保健、休闲等新的消费热点。

　　 （三）全面推进各项改革，力争在体制创新方面走在前列

　　 全面正确地把握、落实党的十六大关于毫不动摇地巩固和发展公有制经济，毫不动摇地鼓励、支持和引导非公有制经济发展的精神。改革、完善国有资产管理体制，积极探索有效的国有资产经营体制和方式。按中央的部署，建立权利、义务和责任相统一，管资产和管人、管事相结合的国有资产管理体制，认真落实国有资本出资人职责。继续推进国有资产授权经营工作。进一步完善公司治理结构，在国有企业董事长由组织考察任命、总经理由董事会聘任、完善企业董事会人员构成、探索实施独立董事制度、强化监事会的独立性、实行外派监事、"一监多企"制度等方面，抓紧研究制定具体实施办法。大力压缩国有企业管理链条，撤销纯管理型集团公司，削减三级企业法人，精减管理机构和领导职数，实行企业几套班子成员交叉任职，有些国有独资企业可不设董事会。加大对国有企业的监管力度，改革完善国有企业领导干部考核办法。按照"有进有退"的原则和"发展壮大一批、整合提高一批、放开搞活一批、关闭破产一批"的思路，加快国有企业产权主体多元化步伐，大力发展混合所有制经济，积极推进国有经济布局的战略性调整。积极引导包括外资、民营资本在内的各类社会资本参与国有企业改革、改组和改造。继续开展大型国有企业产权转让国际招标改革试点工作。选择一批国有独资或国有控股企业，在产权交易中心公开挂牌或在网上招标进行产权转让。积极推行企业经营者和员工持股，鼓励企业管理层收购企业产权。加大市属国有上市公司重组力度。在全市范围大力推动国有企业的资产、业务重组和专业化经营，组建、培育一批具有较强竞争力的大企业集团。深化集体企业改革，继续支持、帮助多种形式的集体经济加快发展。

　　 积极鼓励、支持和引导民营经济加快发展。对民营企业实行政治平等、政策公平、法律保障、放手发展的方针，切实为民营企业的创业者和从业人员营造社会上有地位、政治上有荣誉、经济上有实惠的良好发展氛围。以提高民营经济整体素质和竞争力为核心，以科技创新、制度创新和管理创新为动力，以建立现代企业制度和现代生产经营方式、实现诚信守法的规范经营为方向，促进我市民营经济的大发展。政府设立中小企业和民营企业服务机构，加强对中小企业和民营企业的引导服务。全面清理和废除影响、限制民营经济发展的各种规定，除国家明确限制的领域外，所有投资领域都要以对国有资本或外资同等的条件对民营资本开放；在实际工作中，不得对民营企业设置进入市场的各种人为障碍。重点指导、支持、帮助一批民营企业做大做强，培育龙头民营企业。加大对民营经济的融资支持，鼓励多渠道筹集担保资金、多形式建立担保机构；扶持和推荐符合条件的民营企业发行债券和到国内外上市融资。建立为民营企业提供技术支持、管理咨询、信息服务、市场开拓和人才培训等方面服务的社会化服务体系。引导民营企业入园区发展，走园区经济之路。依法保护民营企业的合法财产和权益。

　　 加快推进行政体制创新，营造良好的政务环境。在坚持我国基本政治制度的前提下，进一步改革政府行政管理体制，从依法规范行政、高效优质行政、民主透明行政和清正廉洁行政四个方面推进具体制度和机制建设，努力建立适应社会主义市场经济体制和世贸组织规则要求，行为规范、运转协调、公正透明、廉洁高效的行政管理体制，把政府职能切实转变到经济调节、市场监管、社会管理和公共服务上来。今年要在中编办指导下，按照精简、统一、效能的原则和行政决策、执行、监督三者既相对分离制约、又协调高效运转的要求，调整政府机构，合理配置政府各部门的职责权力，探索建立现代行政组织结构和运行机制。进一步深化审批制度改革，巩固和提高政府行政效率"提速"成果，在市民中心设立政务综合服务大厅，方便基层单位和市民办理各类审批事宜。加快电子政务建设，建立市、区两级政府及各部门互联互通的信息网络平台，完善面向社会的政府信息服务系统和办事系统。认真落实市政府《关于调整市区两级政府部分管理权限的意见》，把部分经济管理和城市管理的权限下放给各区政府。按照政事分开和精简机构、人员的原则，分类推进事业单位改革。

　　 下大力气推进投融资体制改革，有效激活民间投资。进一步开放投资领域，降低市场准入门槛，通过多种方式有效引导民间资本、外资投向基础设施和基础产业以及教育、文化、医疗、体育等领域，并建立公用基础设施项目建设监管机制。改革社会投资项目审批制度，按照产业政策导向，推行登记备案制。今年力争实施轨道交通、高新技术产业带、物流园区、环境保护、污水处理、文化和医疗项目等领域的投融资体制改革。利用世行资金搞好河流污染治理。加强政府与银行、银团的合作，用好国家政策银行的中长期贷款，并积极探索发行债券，充分利用资本市场。

　　 认真落实"文化立市"战略，加快文化体制改革步伐。进一步完善和组织实施"深圳市文化体制改革总体方案"。政府有关部门要进一步转变职能，从以办文化为主向以管文化为主转变，从以管理政府文化机构和设施为主向管理全社会文化转变，从以行政手段管理为主向以法律和经济手段管理为主转变。对文化事业单位实行分类改革，大力发展公益性文化事业，增加群众性文化活动场所，适当资助准公益性文化事业，加快非公益性文化事业市场化步伐。积极稳妥地推进文化领域对内、对外开放，鼓励非国有经济参与文化建设。研究制定文化产业发展规划，做大做强我市文化产业。坚持集团化、集约化发展，完善深圳报业集团管理体制和经营机制，抓紧组建深圳广电集团、发行集团和演艺集团。做好"中国（深圳）国际文化产业博览交易会"和"深圳国际音乐节"的筹办工作，多出文化精品力作，并以大型文化节庆和重大文化活动为载体，不断丰富人民精神生活，扩大深圳文化影响，提高我市文化产业的竞争力。

　　 （四）扩大对外开放和对内合作，积极拓展发展空间

　　 抓住当前世界经济结构调整的大好机遇，加大优化投资环境与招商引资工作力度，并把引进外资与优化经济结构有机结合起来，提高利用外资的质量和水平。吸引更多跨国公司向深圳转移高新技术产业和技术含量高的大制造业，设立研发中心和区域总部。继续做好服务贸易领域对外资扩大开放的工作，积极创造条件、做好工作，争取一批世界知名企业进入，提升我市服务贸易业的水平。积极探索运用收购兼并、风险投资、投资基金等新的利用外资形式，以及招商引资的新方式、新机制。采取有力措施促进外资企业增资扩产和"三来一补"企业转型升级，扎根深圳。外事、侨务、台务工作要在对外开放中发挥更大作用。

　　 加强区域经济合作，扩大区位优势。把握我国内地与香港构建更紧密经贸关系的机遇，按照"着眼全球竞争，立足两地双赢，携手优势互补，共谋比翼齐?"的思路，积极推进深圳与香港合作向更广领域、更高层次发展。继续加强深港跨境大型基础设施项目建设和口岸通关方面的合作，加快推进西部通道、铜鼓航道、皇岗-落马洲新建公路桥及人行通道桥和罗湖、皇岗、沙头角、文锦渡口岸改造等工程。加快推进深港两地机场的合作，积极推进海港空港运输和物流、金融、旅游、中介服务等现代服务业领域的合作。加强高科技领域的合作，共同培育深港科技风险投资市场体系和科技服务中介体系，研究探讨深港接壤地区合作开发问题。在粤港合作联席会议框架下，推动建立深港两地政府高层间的磋商、协调工作机制。积极与香港联手到欧、美、日、韩等国家和地区联合招商引资，推介旅游和贸易。在省委、省政府领导下，加强与珠三角各城市及省内其他城市的合作，全面提升经济合作水平，实现区域资源整合和优势互补、共同发展。加强与我国广大中西部地区的合作。坚决完成好国务院、省下达的对口帮扶任务，把对口帮扶与经济合作有机结合起来。鼓励支持企业遵循市场经济规律积极参与西部大开发，到中西部地区投资设厂、兼并收购企业、拓展服务业，形成以深圳为基地、辐射中西部地区的生产组织体系和服务体系。继续发挥好内地驻深机构的桥梁纽带作用。

　　 （五）继续优化投资发展环境，进一步提高我市综合竞争力

　　 采取有力措施，继续控制、降低营商成本。强化监管力度，保证降低行政事业性收费的各项规定落到实处。积极推进降低经营服务性收费的工作。在全市范围全面实施撤销路桥收费站、降低路隧收费（除高速公路外）的改革措施。争取进一步降低销售电价。适度控制土地和房地产价格水平。

　　 强化市场监管，规范市场经济秩序。加大体制机制创新和法治建设力度，营造与WTO规则相适应、与国际惯例接轨的市场环境。继续开展专项整治，坚决打击各种破坏市场经济秩序的行为。不断完善有形建筑市场、土地交易市场和产权交易市场建设。加大知识产权保护力度。搞好税法宣传，强化税收征管，加快推进税收信息化建设，严厉打击偷、逃、骗税行为。继续加强社会信用制度建设，完善企业和个人两个信用信息征集系统，积极发展资信评估业。整顿规范中介组织，加快行业协会的改革发展，努力培育一批素质高、公信力强、公平竞争的中介组织，建立健全社会经济监督服务体系。继续抓好清理违规、逾期借款和违规担保贷款工作。

　　 加大人才培养和引进力度，构筑人才高地。牢固树立人才是第一资源的观念和海纳百川的人才观，全面实施人才发展战略，逐步实现人才特别是高级人才由引进为主向引进与自主培养并重的转变。以深圳大学、深圳职业技术学院和大学城为依托，着眼于产学研的紧密结合、科技与经济的紧密结合，广泛利用国内外教育资源，走高等教育跨越式发展之路。通过公办、民办、外资办等多种形式办学，规划建设若干所以培养高级技工、技师为主的高等职业技术院校。健全和完善人才引进政策，创新人才引进机制，大力引进国内外高素质人才。改革人事制度和分配制度，完善生活配套设施，营造吸引人才、留住人才、用好人才、爱护人才的良好环境。

　　 研究探索在WTO规则框架下如何有效实施宏观调控政策，营造良好的政策环境。设立产业发展基金，用于支持高新技术和现代服务业等产业的发展，研究制定和完善促进外贸出口的各项扶持政策，努力保持政策的连续性和稳定性。试行减轻跨国公司采购中心和研发中心外籍人员税负。

　　 （六）坚持可持续发展战略，努力提高城市规划、建设、管理的现代化水平

　　 适应城市发展新形势，重点解决城市规划、建设、管理中的突出问题。要以国内、国际先进水平为标杆，积极探索建立规范高效的现代城市管理体系，逐步实现城市管理的法治化、科学化、信息化，切实提高城市管理现代化水平。一是按可持续发展的要求，做好新一轮城市规划的编制、修订与细化工作，切实发挥城市规划在城市发展中的龙头作用，保持规划的先进性和前瞻性，充分发挥城市规划对经济社会发展和城市建设的综合调控作用。完成全市近期建设规划和2030年城市发展策略研究。完善、调整各区的城市规划。二是努力加快宝安、龙岗两区的城市化进程，提高其城市化水平，促进特区内外协调发展。特区外要加大基础设施建设投入，高标准搞好卫星新城规划建设，强化和完善镇村规划建设。要在充分调研基础上，选择条件成熟的镇、村进行撤镇建街、撤村建居委会的改革试点，加快推进农村城市化进程。特区内要做好股份合作公司的改造和发展工作。三是切实加强人口调控，研究调整招调迁户和暂住人口管理政策，控制人口总量的过快增长，优化人口构成，提高户籍人口比例。四是深入开展交通综合整治，使群众出行方便快捷。加强路网规划建设，挖潜改造、疏?"瓶颈"，提高次干道和支路网密度。大力提高交通管理水平，确保各交通"瓶颈"、重点路段、重点时段车路畅通。合理调整公交线路，搞好公交站点、枢纽站、停车场和人行天桥的规划建设，有效缓解深南大道等路段公交车排长龙的现象。加强道路交通管理和交通安全法制建设，增强驾驶员和行人遵守交通规则的自觉性。五是加强土地管理，引导房地产业均衡有序、健康适度发展。对全市建设用地进行严格的总量控制，把好土地供应源头，健全土地储备制度。将存量土地和旧城改造项目纳入土地供应计划管理，引导其逐步释放。提高政府对房地产市场的宏观调控能力，进一步搞好房地产市场预测和监控。加强房地产规划管理，深南大道、滨海大道、北环路等主要交通干道路边第一排和新的市中心区不再批准住宅项目。停止执行购房入户政策。六是进一步加大违法建筑的查处力度，及时办理历史遗留违法建筑的手续，坚决拆除1999年3月5日市人大有关法规文件出台以后抢建的违法建筑，落实属地管理和日常巡查责任制，杜绝新的违法建筑。要将违法建筑的查处与旧城、旧村改造结合起来，加快解?"插花地"、"城中村"、"烂尾楼"等问题，进一步改善城市面貌。

　　 强化城市环境管理，加强生态环境保护。建立长效机制，下大力气搞好市容环境综合整治。加强市容环境管理的宣传教育和法制建设，增强市民群众共同维护良好市容卫生的自觉性。建立道路清扫作业招投标和淘汰制度，切实提高城市保洁水平和质量。继续做好城市管理综合执法工作，加强执法队伍建设，完善城管行政执法与公安协同配合的机制。充分发挥区、镇（街道）、居委会、小区管委会和村在市容环境综合整治及日常管理中的基础性作用，落实管理责任制?"门前三包"保洁责任制。努力提高城市绿化率和绿化水平，扎实完成每年植树500万株的任务，加快园博园和一批市政公园、郊野公园建设，积极推进生态风景林建设。加强环境综合治理和环境保护，促进城市生态环境的良性循环。认真落实污染物排放总量控制计划，完善污染源监督管理长效机制，实现工业污染源全面达标。加快污水排放网建设，做好生活污水的净化处理。高度重视解决河流污染问题，积极开展水环境治理工作，年底龙岗河、坪山河要基本达到农业用水要求，观澜河、深圳河污染趋势要得到有效遏制，其他河流和水资源保护区的污染治理工作要按计划扎实推进。切实做好大气污染、噪声污染的防治工作，加强对机动车尾气排放的监测控制。继续搞好水土保持，加大裸露山体和采石场、取土区的综合整治工作力度。加强海洋生态环境的保护。

　　 （七）加强社会主义精神文明和政治文明建设，大力发展各项社会事业

　　 扎实推进精神文明建设。深入开展邓小平理论和"三个代表"重要思想的宣传教育，把十六大精神的学习贯彻不断引向深入。继续贯彻落实《公民道德建设实施纲要》和《深圳市民行为道德规范》。弘扬新的深圳精神，形成新时期经济特区建设事业的强大精神动力。强化"深圳人"意识，不断提高城市的凝聚力，增强市民的归属感。以创建文明市、文明区为龙头，广泛开展各种群众性创建活动。加强国防教育，做好征兵和民兵预备役工作。巩固和发展军政、军民团结，力争今年再获全国"双拥模范城"称号。

　　 切实加强民主法制建设。各级政府要自觉坚持向人大及其常委会报告工作，自觉接受其监督；充分尊重和支持人民政协、各民主党派、人民团体在政治协商、参政议政和民主监督中的作用；政府重大决策要向人大报告、向政协通报，征询并认真听取人大、政协及各民主党派的意见；切实做好人大、政协各项议案、建议、建议案、提案的办理工作。坚持依法治市、依法行政。加大法制宣传力度，提高公民的法律意识。继续推进政府行政的法定化工作，加强行政执法，建立行政执法主体的公告制度，强化执法监督。继续加强基层民主法制建设，坚持推行并不断完善政务、村务、企务公开和基层民主选举制度。加快推进社区建设。

　　 大力发展各项社会事业。继续实施科教兴市战略，努力建设学习型城市，创建教育强市。加大教育投入，积极推进教育体制创新，规范教育管理。继续扩大我市高等院校招生规模，合理调整学科和专业设置，加强重点学科和重点实验室建设，全面提高办学质量和水平。大学城西校区要于今年8月份建成交付使用。以素质教育为核心，进一步提高基础教育水平，增加学位，扩大优质教育资源，推进标准化和信息化建设，加快示范性高中学校的建设。积极发展职业教育和继续教育，完善终身教育体系。加快哲学社会科学发展，使之更好地服务于我市现代化建设。深化卫生体制改革，全面落实区域卫生规划，进一步改善医德医风，提高医疗质量和服务水平，努力降低医疗费用，继续推进社区健康服务中心建设。加快华南医院等几所高档次医院的建设，搞好人民医院、眼科医院、市中医院的改扩建工程。做好计划生育工作，不断提高计生服务水平。提升竞技体育水平，办好第六届市运会，力争在第五届城运会中取得好成绩。加快公共体育设施的规划建设，广泛开展全民健身活动。大力发展体育产业。档案工作要努力取得新进展。

　　 （八）关心群众生活，维护社会稳定

　　 努力做好就业和再就业工作，切实解决困难群众的生产和生活问题。就业是民生之本，要全面落实促进就业和再就业工作的各项政策，做好居民按比例就业工作，完善劳动就业服务体系，积极开展再就业援助行动。深化住房制度改革，推进安居房和廉租屋制度的实施，建立健全社会住房保障体系。进一步完善覆盖全市的多领域、多层次的社会保障体系。继续做好"一个低保"和"两个确保"工作，做到应保尽保。认真解决困难群众在子女入学、就医、法律援助等方面的实际困难，使每一个困难群众都得到实实在在的帮助。高度重视、积极支持老龄工作和残疾人事业，切实保障妇女和未成年人的合法权益。扎实做好第二期同富裕工程最后一年的工作。

　　 加大维护社会稳定工作力度。坚持不懈地与各种敌对势力及"法轮功"等邪教组织作斗争。强化社会治安综合治理，严厉打击各类刑事犯罪，特别是带有黑社会性质的犯罪团伙。进一步理顺公安管理体制，加强公安队伍建设，提高干警整体素质，增强战斗力。完善报案系统，充实一线警力，强化路面巡逻防控和对流动人口、出租屋的管理，搞好安全文明小区建设，建立健全快速反应的综合群防群治网络，打防结合，以防为主，竭尽全力为广大群众提供良好的治安环境。落实安全生产责任制和行政责任追究制度，狠抓安全生产专项整治和日常监管、预防工作。全面铺开无公害食品行动计划工作，强化食品卫生、药品安全监管力度。完善劳动安全、劳动仲裁和欠薪保障制度，维护劳动者合法权益。深化农村税费改革，切实减轻农民负担。重视民族宗教事务，加强信访工作，充分发挥基层调解组织作用，及时妥善化解人民内部矛盾。

　　 （九）加强政府自身建设，努力构建服务型、效能型、责任型政府

　　 坚持执政为民，努力提高服务水平。我们的权力是人民赋予的，纳税人是我们的衣食父母，为人民群众谋利益、为企事业单位和基层服务，是政府公务员的职责所在。我们要按?"三个代表"重要思想的要求，牢记全心全意为人民服务的宗旨，坚持"权为民所用，情为民所系，利为民所谋"，将"以民为本，民为父母"的理念贯穿到政府工作的各个方面和各个环节中去，自觉为市民服务、为企业服务、为纳税人服务，切实把人民群众的利益维护好、实现好、发展好。坚持政务公开，从体制、机制上提高政府工作的透明度，真正体现人民当家作主的原则。要进一步整顿机关作风，强化服务意识，大力提高政府部门尤其是窗口部门、窗口行业的服务水平和服务质量。对不认真为群众和企业服务、办事拖拉敷衍?"吃、拿、卡、要"、作风蛮横霸道等行政"不作为"和"乱作为"行为，要坚决查处，决不姑息。谁对不起人民群众，我们就对不起谁！

　　 坚持真抓实干，认真推动各项工作的落实。市委三届六次全会和本次人大会议进一步明确了深圳发展的目标定位、发展思路和今年的各项任务，大政方针已定，目标任务明确，关键是要抓好落实。要进一步明确抓落实的工作责任制，建立健全抓落实的督促检查和奖惩机制。政府各级领导干部要把工作重心下移到基层，经常深入基层了解掌握实际情况，及时研究解决具体问题，认真扎实地开展工作。要切实减少会议和文件以及不必要的应酬接待，集中精力抓好各项工作。

　　 坚持艰苦奋斗，勤俭办一切事业。要按照胡锦涛总书记在西柏坡学习考察时重申的"两个务必"的要求，继续保持谦虚、谨慎、不骄、不躁的作风，继续保持艰苦奋斗的作风。近年来，市委、市政府反复强调干部党员的精神状态问题，最根本、最核心的就是要弘扬艰苦奋斗精神。在政府工作中，要厉行节约，珍惜、用好纳税人的每一分钱，坚决制止挥霍公款吃喝玩乐，违反规定或超标准修建和装修楼堂馆所，搞华而不实的"政绩工程"、"形象工程"、庆典活动等种种不良风气。要切实抓好增收节支工作，继续降低各种会议和接待费用，严格控制出差、出国考察费用，制定并认真落实节省开支的具体指标和措施，切实降低行政成本。要扩大政府绩效审计范围，加强对政府投资项目的稽察监管力度，严格概预算控制。继续推进公共财政改革，巩固、拓展部门预算、政府采购、收支两条线等改革成果，加快推进市财政集中支付制度改革。要严肃财经纪律，加强对政府各部门资产和资金的管理。深入开展廉政建设和反腐败斗争，切实做到廉洁从政、干净干事。

　　 各位代表，深圳的发展正处在向基本实现社会主义现代化目标冲刺的阶段，我们肩负的任务光荣而艰巨。创新是深圳的灵魂，是深圳发展的永恒主题和不竭动力。我们要进一步解放思想，与时俱进，继续以勇立潮头、敢闯敢试的气魄和胆略，全面推进理论与观念创新、体制创新、科技创新、产业创新、文化创新、环境创新、管理与服务创新等各方面的创新，坚决破除一切妨碍发展的认识做法，坚决废除一切束缚发展的条条框框，坚决革除一切影响发展的体制机制障碍和弊端，放手让一切劳动、知识、技术、管理和资本的活力竞相迸发，让一切创造社会财富的源泉充分涌流，汇聚于深圳率先基本实现现代化，建设中国特色社会主义示范地区伟大事业的洪流之中！

　　 各位代表，让我们紧密团结在以胡锦涛同志为总书记的党中央周围，在省委、省政府和中共深圳市委的领导下，高举邓小平理论伟大旗帜，全面贯彻"三个代表"重要思想，沿着党的十六大指引的方向，振奋精神，励精图治，开拓创新，扎实工作，在新的起点上努力开创深圳发展的新局面，为我国全面建设小康社会，加快推进社会主义现代化作出新的贡献!
